# The long term effects of early analysis of a trauma registry

**DOI:** 10.1186/1749-7922-4-42

**Published:** 2009-11-24

**Authors:** Sami Shaban, Mazen Ashour, Masoud Bashir, Yousef El-Ashaal, Frank Branicki, Fikri M Abu-Zidan

**Affiliations:** 1 Department of Medical Education, Faculty of Medicine and Health Sciences, United Arab Emirates University, Al-Ain, PO Box 17666, UAE; 2 Department of Surgery, Faculty of Medicine and Health Sciences, United Arab Emirates University, Al-Ain, PO Box 17666, UAE; 3 Department of Emergency Medicine, Al-Ain Hospital, Al-Ain, PO Box 1006, UAE; 4 Department of Surgery, Al-Ain Hospital, Al-Ain, PO Box 1006, UAE

## Abstract

**Background:**

We established a trauma registry in 2003 to collect data on trauma patients, which is a major cause of death in the United Arab Emirates (UAE). The aim of this paper is to report on the long term effects of our early analysis of this registry.

**Methods:**

Data in the early stages of this trauma registry were collected for 503 patients during a period of 6 months in 2003. Data was collected on a paper form and then entered into the trauma registry using a self-developed Access database. Descriptive analysis was performed.

**Results:**

Most were males (87%), the mean age (SD) was 30.5 (14.9). UAE citizens formed 18.5%. Road traffic collisions caused an overwhelming 34.2% of injuries with 29.7% of those involving UAE citizens while work-related injuries were 26.2%. The early analysis of this registry had two major impacts. Firstly, the alarmingly high rate of UAE nationals in road traffic collisions standardized to the population led to major concerns and to the development of a specialized road traffic collision registry three years later. Second, the equally alarming high rate of work-related injuries led to collaboration with a Preventive Medicine team who helped with refining data elements of the trauma registry to include data important for research in trauma prevention.

**Conclusion:**

Analysis of a trauma registry as early as six months can lead to useful information which has long term effects on the progress of trauma research and prevention.

## Introduction

Trauma is the cause of 10% of all deaths worldwide [1] and it is projected that road traffic deaths will increase by 83% between 2000 and 2020 in developing countries [2]. Trauma is a major health problem in the United Arab Emirates (UAE). About 18% of the annual mortality in UAE is due to trauma and most of these deaths are caused by road traffic collisions [3]. Trauma affects mainly the young productive population which has a profound health and economic impact. Prevention of trauma is not only the most effective method of reducing the toll of death but also the cheapest [4].

The first step in planning for trauma prevention is to collect data through trauma registry surveillance systems [5]. Trauma registries are databases that document trauma cases according to specific inclusion criteria [6]. They are designed to improve injury surveillance and enhance trauma care, outcomes, and prevention [4]. It has been shown that trauma registries in developing countries are plausible and valuable tools for injury surveillance [4,5].

One of the major problems of trauma registries is obtaining continuity of funding to ensure the stability of data collection by trained personnel [7]. The strength of registries comes from their ability to follow the progress of trends of studied variables over time [5]. This fundraising difficulty may discourage clinicians and policy makers from establishing registries which may collect data for only a limited period. Our encouraging experience in establishing a trauma registry and the impact of early analysis of the registry data and its long term effects is informative and may be well of widespread interest.

## Patients and Methods

Establishment of the Trauma Registry at Al-Ain Hospital passed through stages:

I. Design of a suitable data entry form: The Trauma Registry form of Trauma Services at Royal Perth Hospital was adopted. It was shortened and modified to fit UAE needs. A working group which consisted of a Trauma Surgeon, an Emergency Physician and a Critical Care Physician was involved in the Development of the Trauma Registry form.

II. Inclusion exclusion criteria were defined after discussion with representatives of the Emergency Department, Intensive Care Unit, General Surgery, and Orthopedics. This registry was limited to those who died after arrival at hospital and for hospitalized patients who stayed more than 24 hours in the hospital. This decision was taken because of limitations in personnel and funding.

III. Suitable computer hardware and software for reliable collection and analysis of data was kindly supplied by the College of Information Technology at the United Arab Emirates University. A database using Microsoft Access program was designed by one of the Authors (SS). Regular discussions helped in the final version of the program. This program was modified after a pilot trial of data entry.

IV. Selection and training of personnel for data entry and analysis: A salary for one year was secured for a research assistant with funding from Research Grant provided by the United Arab Emirates University. A young medical graduate, who was computer literate, was selected to collect and enter data. Data collection began on 15 March 2003 and information entered on the database. Data entry was regularly monitored and the necessary support was supplied to train the research assistant.

Early data analysis of the trauma registry was performed in 2003 for data collected at that time and presented at an international conference [8]. The long term effects of the results of early analysis on our strategic plan in trauma research is reported.

## Results

### Early analysis of data

**Figure 1:**
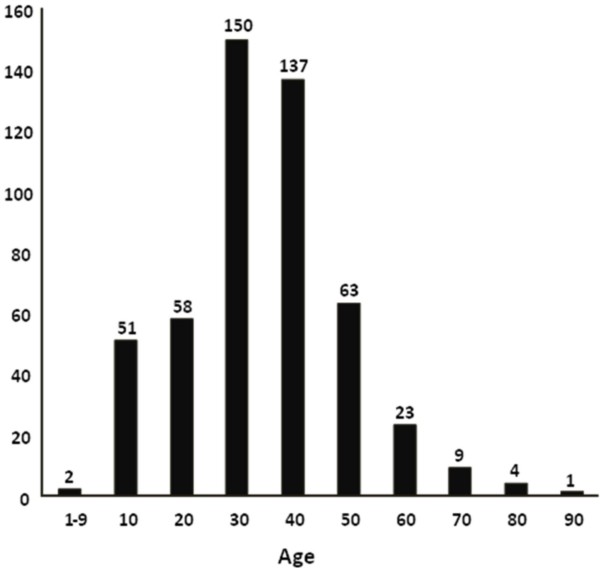
**Age distribution of the study population**.

Five hundred and three patients were registered during the period 15 March 2003 until 15 September 2003. 439 were males (87%) and 64 females (13%) with a mean age (SD) of 30.5 (14.9) years, and age ranged between 1 and 88. 79 patients were less than 16 years old (15.7%). Age distribution is shown in Figure 1. The four most frequent nationalities of the injured were Pakistani (99, 19.7%), Indian (96, 19.1%), UAE citizens (93, 18.5%) and Bangladeshi (50, 9.9%). Thirty nine patients (8%) were admitted to the Intensive Care Unit (ICU). One hundred and thirty two (26.2%) were work related injuries. Patients stayed a mean of 9.6 days in the hospital. Nine patients (1.8%) who arrived alive at the hospital eventually died in hospital. Road traffic collisions caused an overwhelming 34.2% of the injuries. Distribution of cause of injury is shown in Table 1.

**Table 1:** Distribution of causes of injury

Cause	Number of patients	%
Road Traffic Accident	172	34.2
Fall From Height	92	18.3
Fall Down	74	14.7
Burn	27	5.4
Heavy Object	27	5.4
Machinery	22	4.4
Assault	20	4
Other	69	13.7

Total	503	100

### Long term effects of early analysis

Firstly, the alarmingly high rate of UAE nationals in road traffic collisions standardized to the population led to major concerns and to the development of a road traffic collision (RTC) registry three years later. This stemmed from a combined effort of the Trauma Group and Preventive Medicine Department to raise funds to develop a specific registry studying the mechanisms of RTCs and use of safety devices with detailed information of RTCs on a sound database. The RTC registry led to a better understanding of road traffic collisions and their impact on the country [9,10].

Secondly, the equally alarming high rate of work-related injuries led to collaboration with a Preventive Medicine team who helped with refining of data elements of the trauma registry to include data important for research in trauma prevention [11-13]. This also led to an understanding that ongoing involvement of researchers with specific interest in community medicine is an essential component of trauma prevention. The trauma registry helped to promote trauma awareness and management in the minds of clinicians.

As a result of collaboration with Preventive Medicine specialists, the registry was modified to contain important information on injury prevention. In addition, several unnecessary variables related to management were removed. Furthermore, and as a result of extensive user needs analysis, the registry interface was also redesigned to be easier to navigate and more user-friendly in general. For data entry, the tabbed design was used to categorize related items of interest and this has proven to be the quickest and easiest data entry method. The relational database design was rechecked and modified accordingly.

## Discussion

We were able to establish a Trauma Registry at Al-Ain Hospital. This was possible with support a research grant from the UAE University. Trauma registries need to be an integral part of health informatics data collection. Such Registries are valuable tools for identifying considerations that require implementation of quality improvement policy and are essential for much needed progress in the health care system [14].

Our early analysis has shown that road traffic collisions caused 34.2% of the injuries while work-related injuries were responsible for 26.2% which has helped us to focus on these two important areas in our community. Several detailed analyses have emerged later from the registry related to RTC or occupational injuries.

A study based on the RTC registry data regarding the driver's pre-incident behavior and mechanism of injury defined the seatbelt compliance to be very low (25%). Front impact and rollover collisions were the most common mechanisms of injury, and only 16% of the drivers were distracted prior to having the crash [10]. Another RTC analysis on factors affecting mortality in RTC found that head injury is the major factor affecting mortality, followed by injury severity and hypotension [9]. Lessons learned for prevention included the need for enforcement of law on seat belt and helmets use in our community.

Occupational injury in the UAE was addressed in a study with collaboration with occupational medicine researchers [13]. The analysis sought to investigate the epidemiology of occupational injury hospitalizations using data from the trauma registry. The incidence of occupational injury hospitalizations was approx 136/100,000 workers/year with 98% being males and 96% being non-nationals. The study concluded that external causes were proportionately much more frequently encountered than in industrialized countries and that effective counter measures are needed to reduce the incidence and severity of these occupational injuries.

Countries with limited resources have been able to establish useful Trauma registries [4,7,15]. Ongoing funding and dedicated personnel are essential for the success of a trauma registry whose staff should be considered as key members of the trauma team. Orientation and training of trauma registry personnel is essential as well as identifying informatics experts to develop and enhance the registry program and analyze the registry data [16].

Trauma registries are useful for collecting continuous, standardized, large sets of data for analysis and enhancing quality of care, ensuring appropriate resource allocation, and offering evidence of trauma incidence and care [4]. This provides more reliable information regarding risk factors related to different types of injuries and ways to prevent them [6]. Furthermore, the merging of trauma registry data with other sources of information related to the injured victims can produce a more descriptive resource [5].

Obtaining research funds for such projects can be very difficult. In our case, the results of early analysis of data was crucial for convincing potential grantors that this type of project is worthwhile and persuading researchers that this is a valid form of Health Informatics research. The next step is to establish a nationwide Trauma Registry using the general web-based database-driven model. This will allow the possibility of combining data from different hospitals and distant regions. Patient data security and privacy are issues that must be dealt with when developing such remote data entry models [16].

A study comparing seven national trauma registries concluded that successful trauma registries show continuous growth of datasets and provide basic data for publications and for policy guidelines [5]. Although, the trauma registry established in 2003 in Al-Ain city, UAE collected data for a finite period of time, it has successfully provided basic data for publications and for policy guidelines. Since the inception of the trauma registry interest in trauma in the UAE has risen dramatically. Collaboration between clinicians, health Informaticians, and preventive medicine specialists has produced a number of publications based on the registry data [8-13,17-20].

## Conclusion

Establishing a Trauma Registry where none has previously existed in a developing country is a challenging task. Nevertheless, it is feasible and has the potential to be developed to a nationwide database. Analysis of a trauma registry as early as six months can lead to useful information which has the potential for long term effects on the progress of trauma research and prevention.

## Competing interests

The authors declare that they have no competing interests.

## Authors' contributions

Sami Shaban helped in the idea and design of the trauma registry form and modified it, designed the electronic trauma registry, analyzed the data, and wrote the manuscript. Mazen Ahsour helped in the idea, collected the data and entered it, and approved the final version of the paper. Masoud Bashir helped in the idea, design of the form, data collection, and approved the final version of the paper. Youref El-Ashaal helped in the idea, design of the form, data collection and approved the final version of the paper. Frank Branicki helped in the idea and design of the form, edited the first draft of the paper and approved its final version. And finally, Fikri M Abu-Zidan had the idea, raised funds for the study, designed the trauma registry form, trained the research fellow for data collection, assured the quality of data collected, did the primary analysis, helped draft the first version of the paper, repeatedly edited it, and approved its final version.
